# Supplementary material for: H2A.X promotes endosperm-specific DNA methylation in Arabidopsis thaliana
Source: BMC Plant Biol. 2023 Nov 22;23:585. doi: 10.1186/s12870-023-04596-y (PMC10664615; doi:10.1186/s12870-023-04596-y)

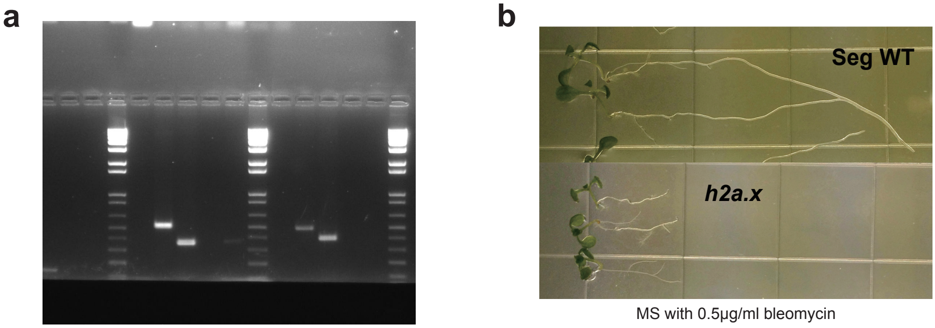


**Figure S1. The full-length gel of Fig. 1b and Increased sensitivity to bleomycin in *h2a.x* mutant.** **a** The complete image depicting qPCR analysis of each mutant, demonstrating cDNA-specific PCR amplification and the absence of the gene product in mutant seedling tissue. **b** Aberrant root growth of *h2a.x* mutant seedlings were observed when grown in bleomycin MS.

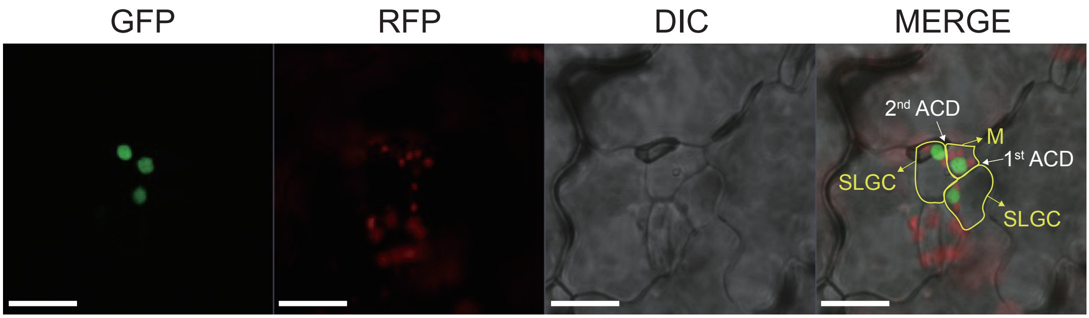


**Figure S2. Stomatal HTA5:GFP expression**
HTA5:GFP is expressed in the adaxial side of the leaf, specifically, in the stomatal linage ground cell (SLGC) and meristemoid (M). ACD, asymmetric cell division. Scale bar, 20μm.

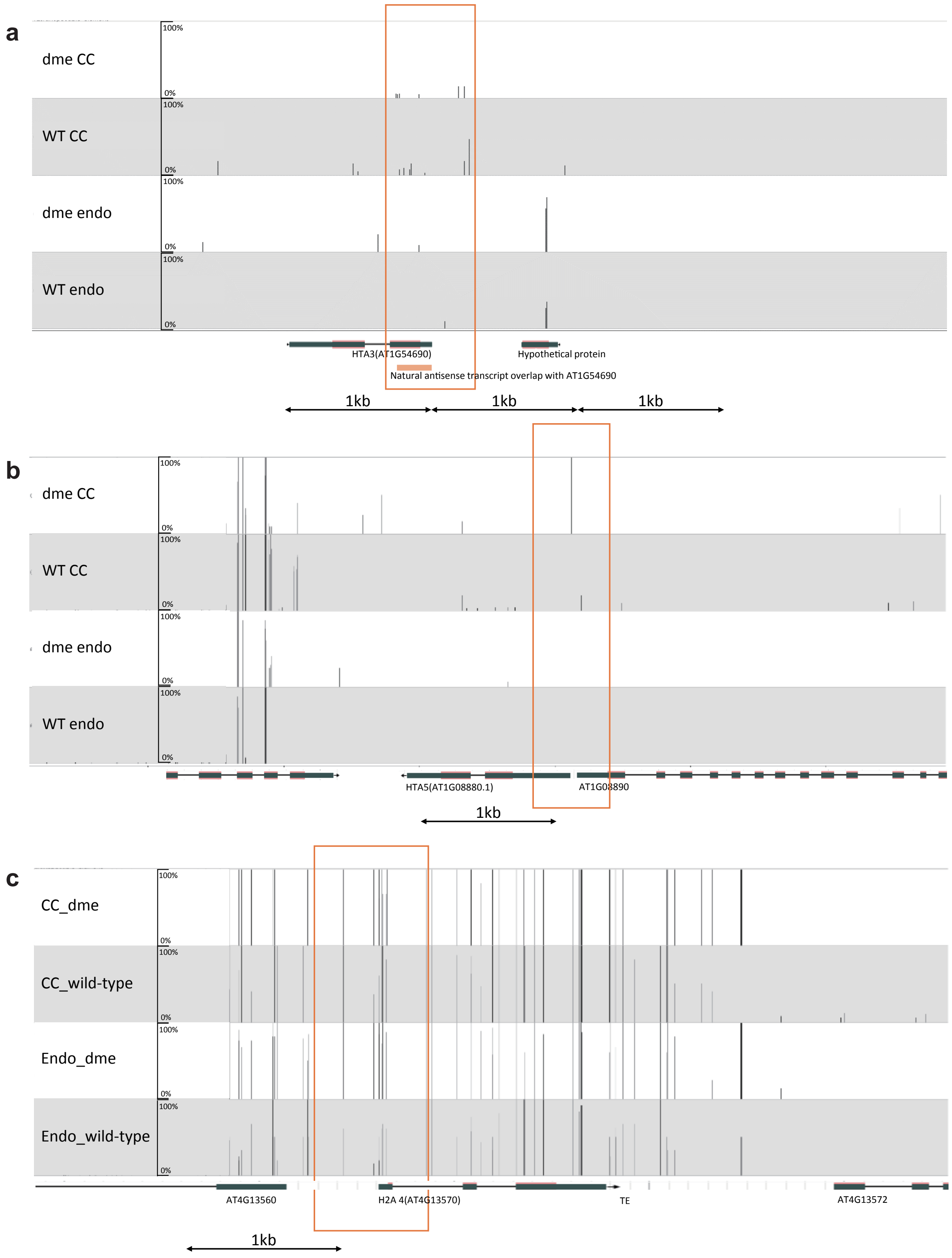

a
dme CC
WT CC
dme endo
WT endo
100%
0%
HTA3(AT1G54690)
Hypothetical protein
Natural antisense transcript overlap with AT1G54690
1kb
1kb
1kb
b
dme CC
WT CC
dme endo
WT endo
HTA5(AT1G08880.1)
AT1G08890
1kb
c
CC_dme
CC_wild-type
Endo_dme
Endo_wild-type
AT4G13560
H2A 4(AT4G13570)
TE
AT4G13572
1kb

**Figure S3. Analysis of CG DNA methylation at H2A variant genomic loci in WT (Col-0) and *dme-2* mutant Arabidopsis.**
**a** HTA3 (H2A.X). **b** HTA5 (H2A.X). **c** HTA4 (H2A.Z). CC = Central cells [24], Endo = Endosperm [11]. Transcription start site and promoter regions are highlighted with orange box. All cytosine methylations are included without read cutoff. All bars of histogram indicate the methylation % level of single cytosine. Bismark was used for bisulphite sequencing data read alignment and Seqmonk was used for visualization.

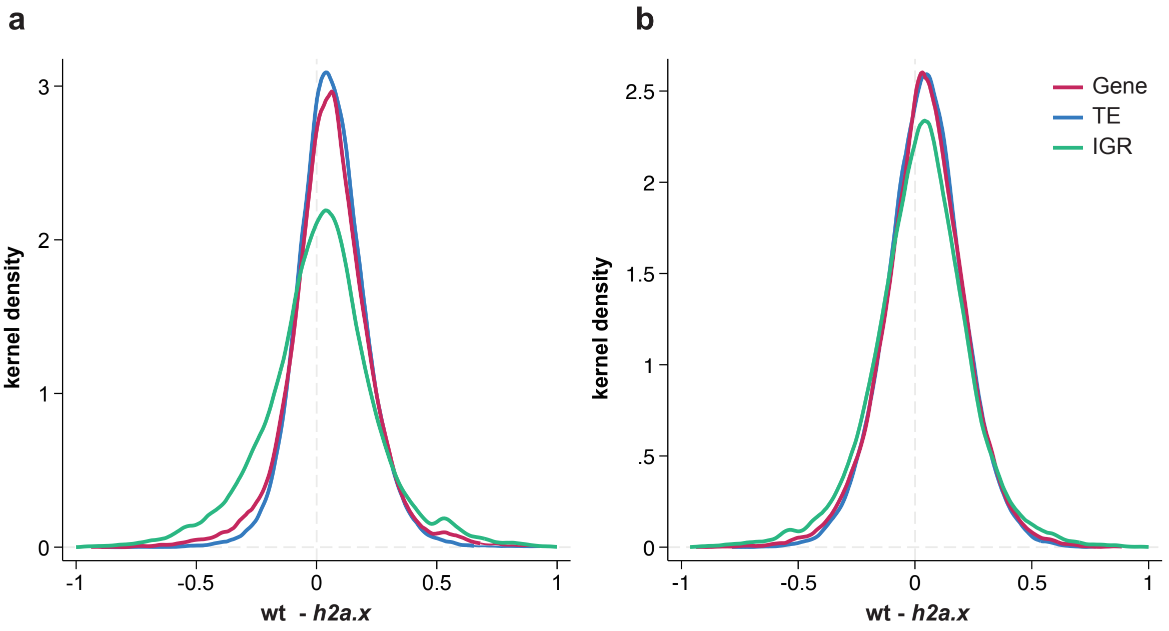


**Figure S4. Methylation analysis in transposon, genic and intergenic genomic contexts, in selfed double h2a.x mutant developing endosperm**

**a** Fractional methylation difference between h2a.x double mutant and WT CG methylation in endosperm is plotted (WT minus h2a.x), data in 50 bp windows with >15x sequence coverage. Transposon (TE) context in blue, Genes in red, Intergenic regions in green. Data are from h2a.x Col selfed plants and segregating WT siblings. All three density plots are centred slightly above zero, indicating relative hypomethylation in h2a.x mutant transposons, genes and intergenic regions in endosperm. **b** As for a, but with wild-type endosperm methylome data from Ibarra et al. Plants are maternal Col-0 crossed to WT Ler though the reads are pooled in analysis. TE context in blue, Genes in red, Intergenic regions in green. All three density plots are again centred slightly above zero, indicating relative hypomethylation in h2a.x mutant transposons, genes and intergenic regions compared to WTmpared to WT

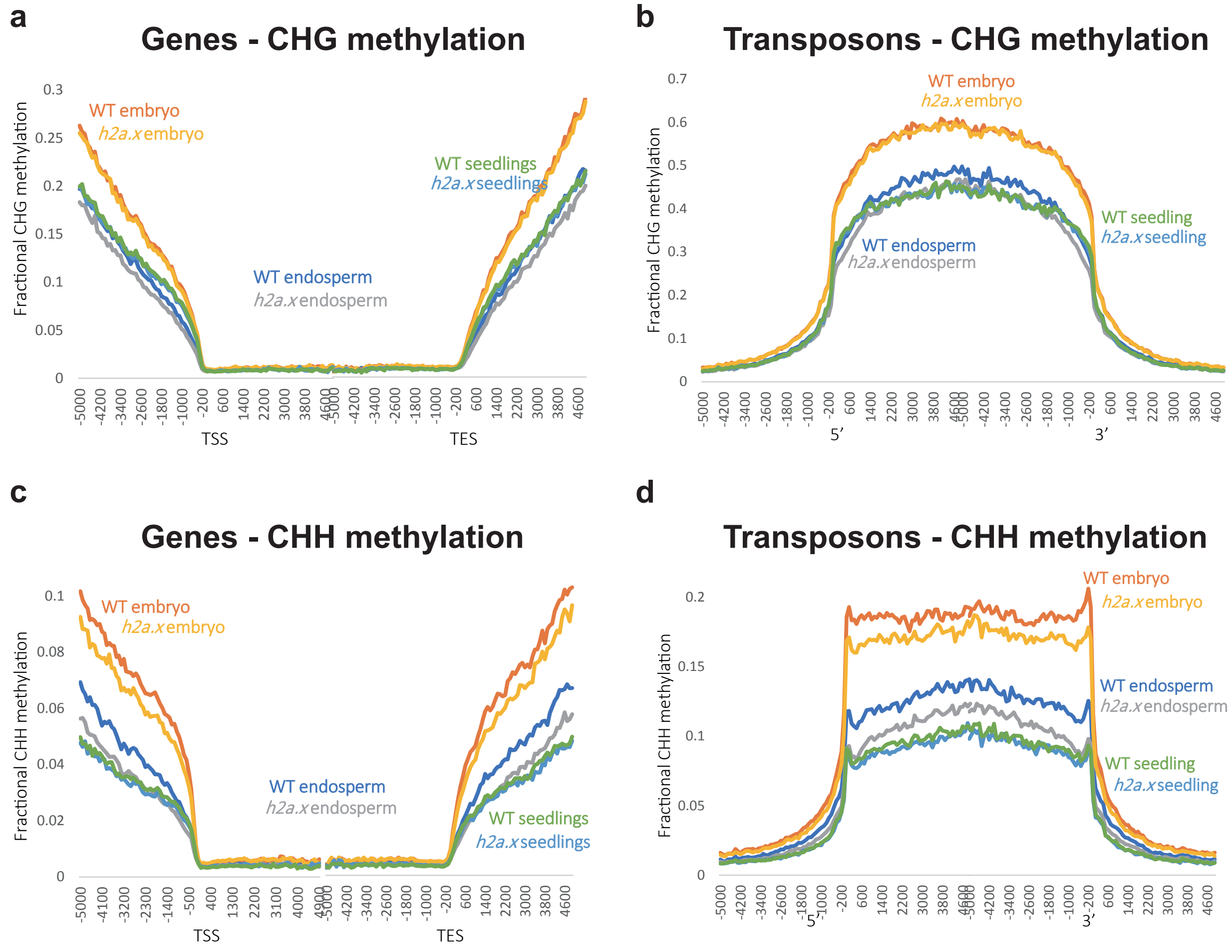


**Figure S5. Non-CG methylation levels Genome-wide CHG and CHH methylation analysis of *h2a.x* mutant developing embryo, endosperm and seedling.**

Ends analysis of selfed *h2a.x* mutant and segregating WT genomic CHG methylation in genes (**a**) and TEs (**b**), as well as CHH methylation in genes (**c**) and TEs (**d**), with those aligned according to their 5' and 3' ends. Data for seedling, endosperm and embryo (linear-bending cotyledon) are shown. Endosperm and embryo are hypomethylated at CHH at gene edges and in TEs. Since embryo CHH methylation levels are incredibly sensitive to gestational age, they may be indicative of slightly later dissection of WT for this sample.

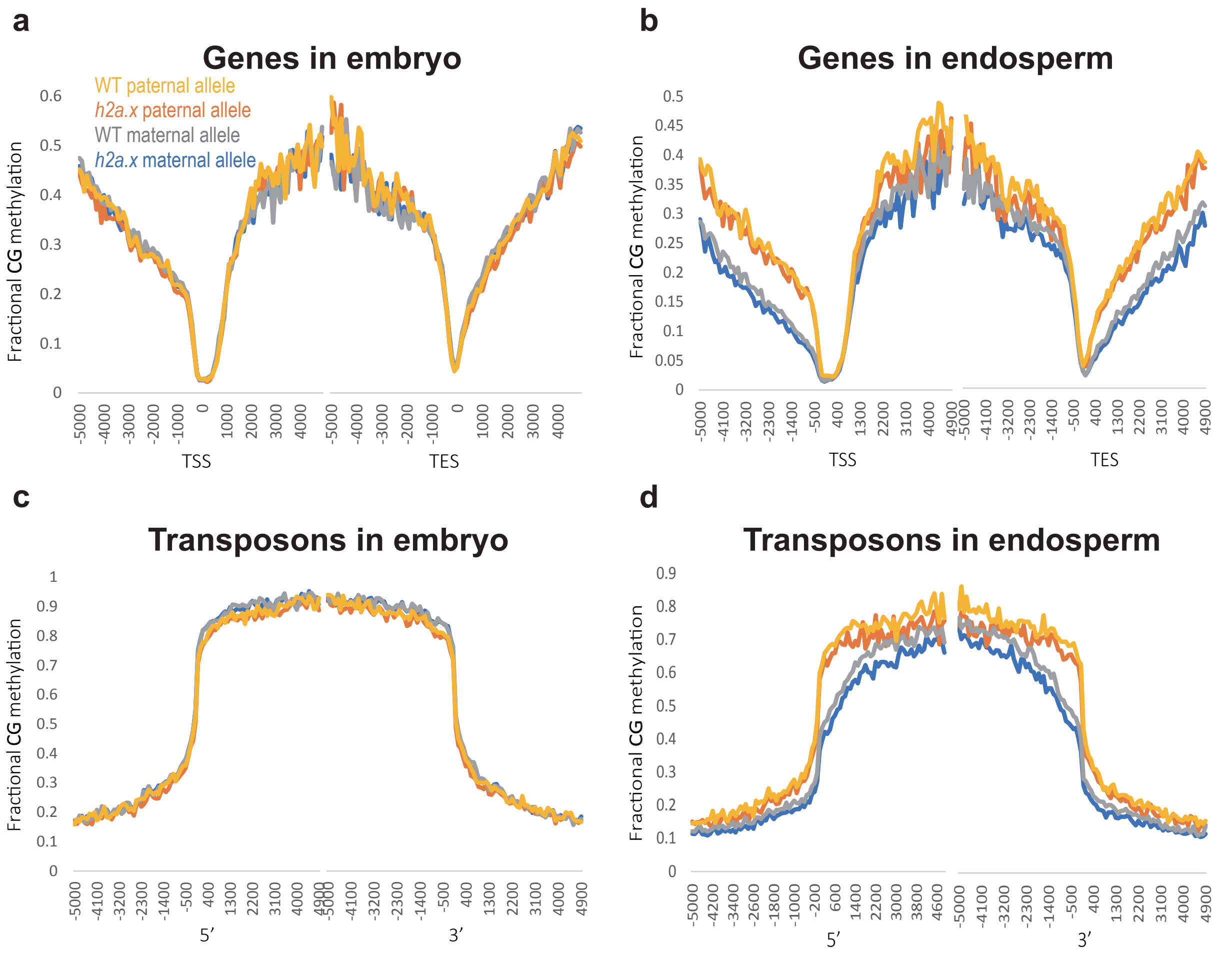


**Figure S6. Parental allele-specific CG methylation analysis of *h2a.x* mutant.**
Female WT Col-0 or *h2a.x* homozygous mutants were crossed with WT Ler pollen and the methylation levels in F1 seeds were analyzed. ‘*h2a.x* paternal’ denotes a WT paternal allele now resident in a heterozygous *h2a.x* mutant seed. Ends analysis of embryo and endosperm CG genomic methylation in genes (**a** and **b**, respectively) as well as those in TEs (**c** and **d**, respectively) are shown, with genes and TEs aligned according to their 5’ and 3’ ends. The maternal *h2a.x* endosperm allele is hypomethylated compared to WT. Ends analysis of embryo and endosperm CHG genomic methylation in genes (**e** and **f**, respectively) as well as those in TEs (**g** and **h**, respectively) are shown, with genes and TEs aligned according to their 5’ and 3’ ends. Ends analysis of embryo and endosperm CHH genomic methylation in genes (**i** and **j**, respectively) as well as those in TEs (**k** and **l**, respectively) are shown, with genes and TEs aligned according to their 5’ and 3’ ends. CHH methylation in genes at intergenic regions is decreased on both *h2a.x* endosperm alleles compared to WT. CHH methylation in TE bodies is decreased on both *h2a.x* embryo and endosperm alleles compared to WT.

**Figure S6 continued.**
**Parental allele-specific CHG methylation analysis of h2a.x mutant.**

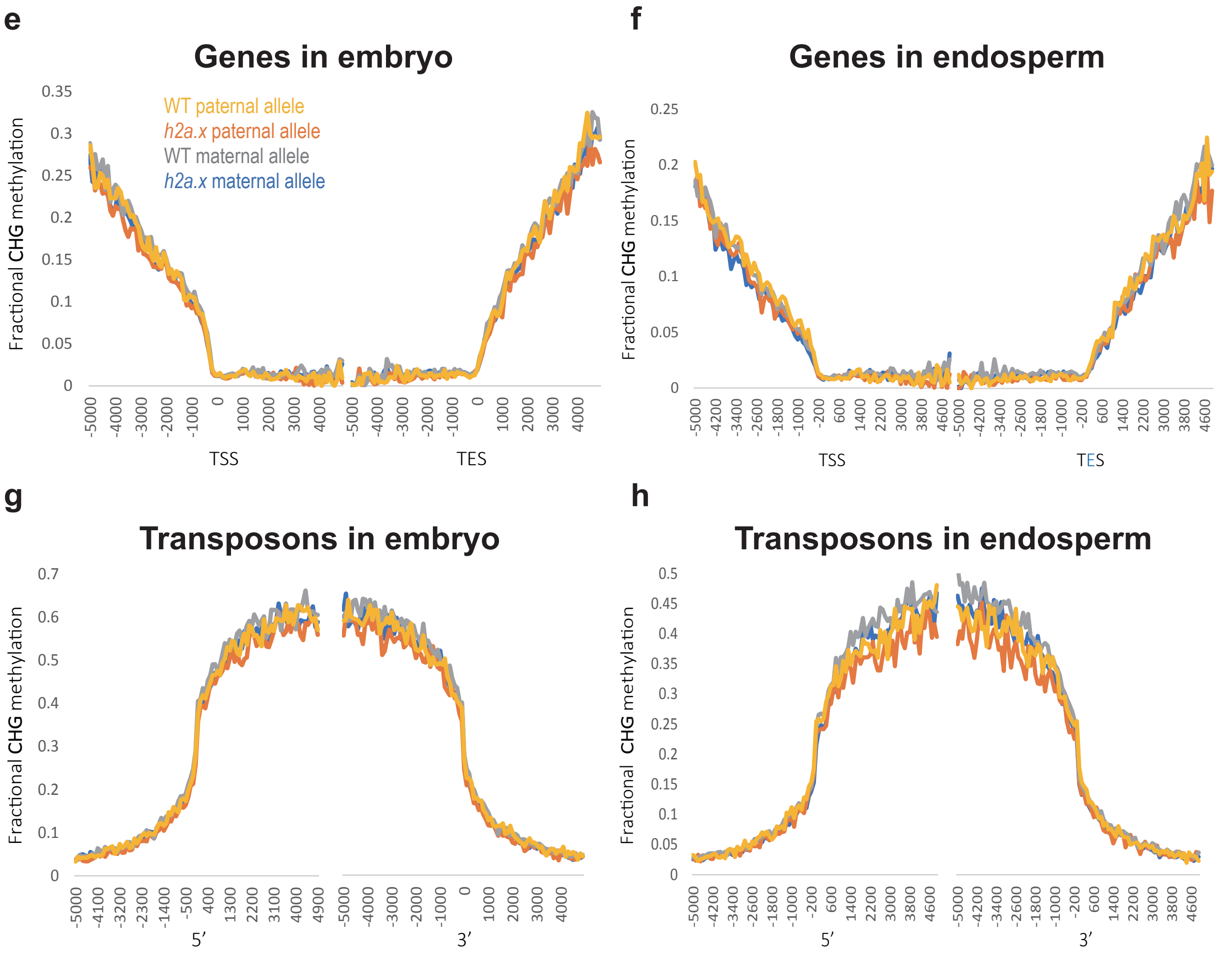

**Figure S6 continued.**
**Parental allele-specific CHH methylation analysis of h2a.x mutant.t**

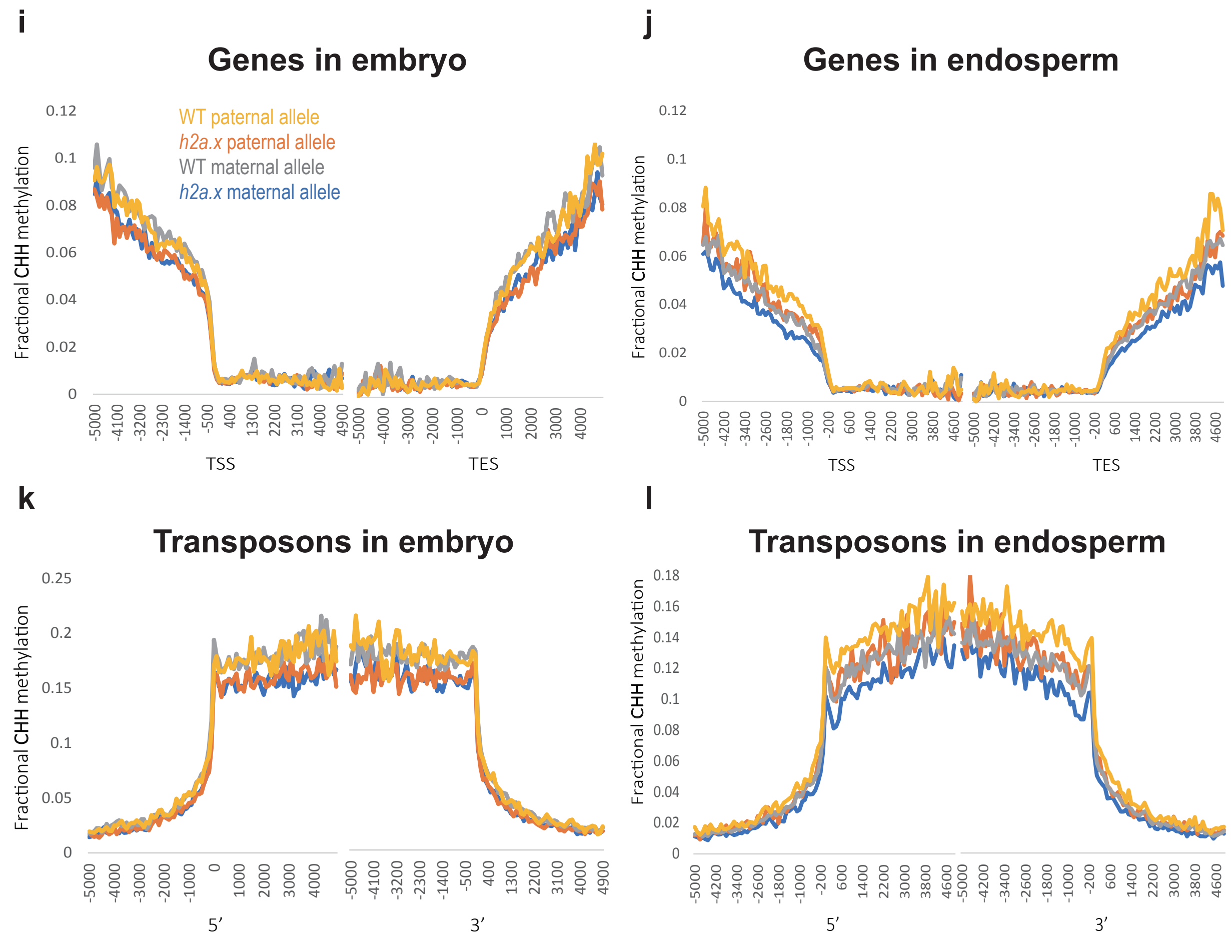

Supplement: Supplementary file 1 — Additional file 1: Fig. S1. The full-length gel of Fig. 1b and Increased sensitivity to bleomycin in h2a.x mutant. Fig. S2. Stomatal HTA5:GFP expression. Fig. S3. Analysis of CG DNA methylation at H2A variant genomic loci in WT (Col-0) and dme-2 mutant Arabidopsis. Fig. S4. Methylation analysis in transposon, genic and intergenic genomic contexts, in selfed double h2a.x mutant developing endosperm. Fig. S5. Non-CG methylation levels Genome-wide CHG and CHH methylation analysis of h2a.x mutant developing embryo, endosperm and seedling. Fig. S6. Parental allele-specific CG methylation analysis of h2a.x mutant. [file 12870_2023_4596_MOESM1_ESM.pdf]
