# Supplementary material for: H2A.X promotes endosperm-specific DNA methylation in Arabidopsis thaliana
Source: BMC Plant Biol. 2023 Nov 22;23:585. doi: 10.1186/s12870-023-04596-y (PMC10664615; doi:10.1186/s12870-023-04596-y)
Supplement: Supplementary file 2 — Additional file 2: Supplemental Table S1. H2A.X genes expression in wild-type and dme endosperm. Supplemental Table S2. BS-seq dataset information. [file 12870_2023_4596_MOESM2_ESM.docx]

**Supplemental Table S1. H2AX genes expression in wild-type and *dme* endosperm**

**Supplemental Table S2. BS-seq dataset information**
